# Leveraging natural language processing and geospatial time series model to analyze COVID-19 vaccination sentiment dynamics on Tweets

**DOI:** 10.1093/jamiaopen/ooad023

**Published:** 2023-04-12

**Authors:** Jiancheng Ye, Jiarui Hai, Zidan Wang, Chumei Wei, Jiacheng Song

**Affiliations:** Feinberg School of Medicine, Northwestern University, Chicago, Illinois, USA; Department of Engineering, Johns Hopkins University, Baltimore, Maryland, USA; Department of Statistics, Northwestern University, Evanston, Illinois, USA; Department of Statistics, Northwestern University, Evanston, Illinois, USA; Department of Diagnostic Radiology, University of Hong Kong, Hong Kong SAR, China

**Keywords:** natural language processing, Bidirectional Encoder Representations from Transformers, sentiment analysis, geospatial analysis, time series, social media, COVID-19, vaccination

## Abstract

**Objective:**

To develop and apply a natural language processing (NLP)-based approach to analyze public sentiments on social media and their geographic pattern in the United States toward coronavirus disease 2019 (COVID-19) vaccination. We also aim to provide insights to facilitate the understanding of the public attitudes and concerns regarding COVID-19 vaccination.

**Methods:**

We collected Tweet posts by the residents in the United States after the dissemination of the COVID-19 vaccine. We performed sentiment analysis based on the Bidirectional Encoder Representations from Transformers (BERT) and qualitative content analysis. Time series models were leveraged to describe sentiment trends. Key topics were analyzed longitudinally and geospatially.

**Results:**

A total of 3 198 686 Tweets related to COVID-19 vaccination were extracted from January 2021 to February 2022. 2 358 783 Tweets were identified to contain clear opinions, among which 824 755 (35.0%) expressed negative opinions towards vaccination while 1 534 028 (65.0%) demonstrated positive opinions. The accuracy of the BERT model was 79.67%. The key hashtag-based topics include Pfizer, breaking, wearamask, and smartnews. The sentiment towards vaccination across the states showed manifest variability. Key barriers to vaccination include mistrust, hesitancy, safety concern, misinformation, and inequity.

**Conclusion:**

We found that opinions toward the COVID-19 vaccination varied across different places and over time. This study demonstrates the potential of an analytical pipeline, which integrates NLP-enabled modeling, time series, and geospatial analyses of social media data. Such analyses could enable real-time assessment, at scale, of public confidence and trust in COVID-19 vaccination, help address the concerns of vaccine skeptics, and provide support for developing tailored policies and communication strategies to maximize uptake.

## INTRODUCTION

In December 2020, the Food and Drug Administration (FDA) in the United States issued the first emergency use authorization (EUA) for use of the COVID-19 vaccine in persons aged 16 years and older for the prevention of coronavirus disease 2019 (COVID-19).^1^ As vaccines against COVID-19 are rolled out, there is a pressing need to better understand and monitor public sentiments and address the concerns of vaccine skeptics. This urgency has been exacerbated by the current situation of the global pandemic and growing pressures on health services. Social media, such as Twitter, has been an appropriate source for understanding public attitudes towards the COVID-19 vaccination.^2^

Artificial intelligence (AI), such as machine learning and natural language processing (NLP), can enable real-time analysis of structured and unstructured data including clinical data^3^ as well as social media data, such as public attitudes, demographic determinants, and popular topics.^4^^,^^5^ This analysis offers the opportunity to track the dynamic public sentiments and develop proactive communication strategies. In addition, an iterative learning cycle based on the analytical process can help identify unforeseen areas of public concerns as well as potential barriers for required interventions, thus maximizing vaccine uptake and minimizing health care disparities across demographic communities.^6^ During the pandemic, NLP has been applied to address many clinical and health care problems. NLP was shown to be useful for extracting signs and symptoms of COVID-19 from clinical notes,^7^ and extracting risk factors related to severe or nonsevere COVID-19 cases from unstructured free text.^8^ NLP has also been used to reveal mental health complaints in real time, recognize vulnerable individuals, and detect rapidly rising mental health-related topics during the COVID-19 pandemic.^9^^,^^10^ The combination of NLP and machine learning algorithms enabled the prediction of potential intensive care unit (ICU) admissions from the electronic health records (EHRs) of patients with COVID-19.^11^ Studies have shown that NLP application on social media data could build useful models to understand health behaviors during the pandemic.^12^^,^^13^ NLP methods were also combined with other computational approaches, such as complex networks, to discover hidden patterns and differences between the communities involved in spreading misinformation and promoting accurate information during the pandemic.^14^

In 2019, WHO named vaccine hesitancy as one of the top 10 threats to global health.^15^^,^^16^ The mutable nature of anti-vaccination calls for new modes of analysis to characterize not only the temporal features of hesitancy but also the spatial (eg, local, regional, national, or international) features and their effects on vaccine uptake. The real-time data on social media also allow investigation into contextual events that can help us understand the barriers to vaccination. This study will leverage a multi-level and integrated analytical pipeline, which includes NLP-enabled modeling, time series, and geospatial analyses of social media data. We will provide a comprehensive analysis of the attitudes of citizens located in the United States toward the COVID-19 vaccination.

This study aims to analyze the Tweet data after the COVID-19 vaccine deployment from January 2021 to February 2022 and answer three questions: (1) What is the sentiment trend towards COVID-19 vaccination over time; (2) What is the geospatial pattern of the sentiment towards COVID-19 vaccination and over time; (3) What are the popular topics and key barriers towards COVID-19 vaccination?

## METHODS

**Figure 1.**
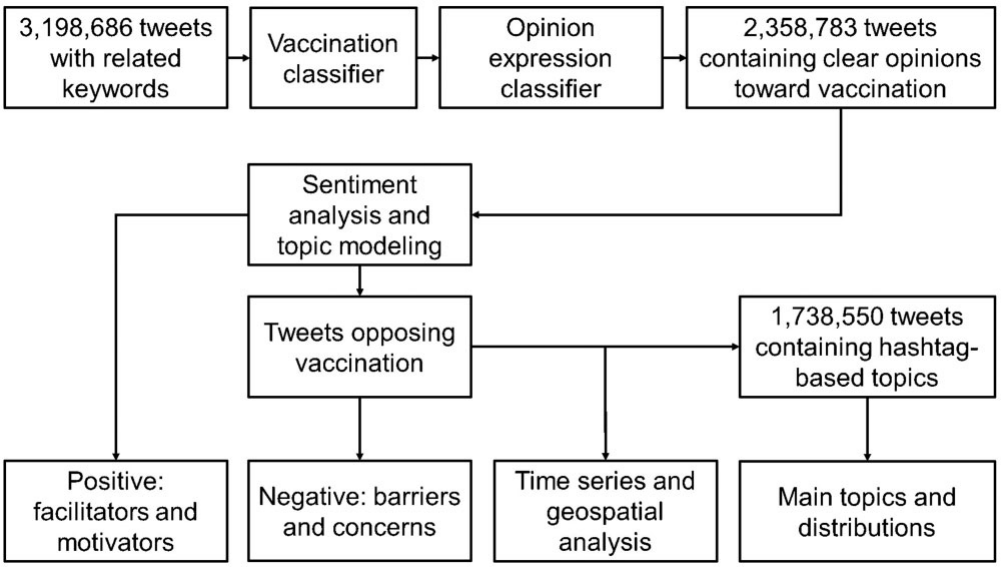
Methods pipeline and flowchart.

This study combined machine and human intelligence to perform the analyses. We collected Tweet posts by residents in the United states from the Twitter Application Programming Interface (API) between January 2021 and February 2022.^17^ The dataset included data collected from the publicly available Twitter Stream API with a collection process that gathered any available Tweets. All the preprocessing scripts utilized components of the Social Media Mining Toolkit (SMMT).^18^ The research team used SMMT to listen to the Twitter Stream API for Tweets with COVID-19 related keywords and then gathered all the Tweets that had the desired keywords before aggregating them locally. The data collected from the stream captured all languages. The analyses in this study were mainly conducted based on the Bidirectional Encoder Representations from Transformers (BERT) model, which was used to explore the main sentiment expressed on the Tweets.^19^ The longitudinal event analysis allowed us to dive a step further into the sentiment pattern over time and explain some of the fluctuations in the time-series results. We also carried out topic modeling focusing on hashtag-based topics. We explored the popular topics from the perspective of sentiment, time series, and geographic pattern, respectively. We also incorporated human intelligence in the analytical process by adding qualitative synthesis of the key barriers and mapping them onto a health care access framework. The overall analytical flow is exhibited in Figure 1.

Since the data analyzed in this study were completely in the public domain, no ethics review was necessary. We conducted a thorough assessment of the privacy risk that our study posed to individuals to ensure compliance with relevant sections of the General Data Protection Regulation.^20^ Further, to comply with privacy laws and social network policies to collect data from Twitter, we did not share or publish personal health information.^21^

### BERT model

BERT is a word representation model that uses unannotated text to perform various NLP tasks such as classification and question answering.^19^ By considering the context of a word using the words before or after, we can produce embeddings for words that are more context-aware. This study used the pretrained sentence BERT model to generate the embedding vectors for emotion classification tasks.

### Sentiment analysis

The sentiment analysis refers to the technique that utilizes NLP and computational linguistics tools to identify or quantify the affective states of the text.^22^ Several theories conceptualize emotional states along two dimensions: valence and arousal.^23^ Arousal is the level of autonomic activation produced by an event and ranges from calm to excited, while valence describes the level of pleasantness and ranges from negative to positive.^24^ For text, arousal states are difficult to recognize, so estimation or classification of valence is the main task of sentiment analysis. Some studies used rule-based approaches such as sentiment dictionaries or traditional supervised classification models such as Logistic Regression, Decision Tree, and Support Vector Machines (SVM) to handle the sentiment analysis task.^25^^,^^26^ However, because these methods could not capture contextual information and interdependence among words, their performances were unpromising when it came to long sentences or complicated contexts.^27^ Later, Recurrent Neural Network (RNN)-based models were widely applied in text and audio tasks to process sequential data.^28^ Recently, with the development of the powerful attention mechanism for dealing with sequential data, transformer-based deep learning models have obtained state-of-the-art results on a range of NLP tasks.^29^ Compared to RNNs, transformers are able to capture long-range dependencies better. Even though transformer-based models are harder to be trained from scratch due to a large number of parameters, publicly available pretrained models, such as BERT and XLNet, enable researchers to easily use transfer learning to train high-performed transformer models on various NLP tasks.^30^

### Hashtag-based topic

Hashtags are central to organize information on Twitter. Designated by a “hash” symbol (#), a hashtag is a keyword assigned to the information that describes a Tweet and aids in searching. Hashtags organize the discussion around specific topics or events. Hashtag use has become a unique tagging convention to help associate Twitter messages with certain events or contexts.^31^ A Twitter hashtag also embodies user participation in the process of hashtag innovation, especially as it pertains to information organization tasks. We leveraged the Latent Dirichlet Allocation (LDA)^32^ to identify and aggregate the main topics from the Tweets containing clear opinions. LDA generates a probability distribution for the text corpus; it assumes that each topic can be characterized by a distribution of words. To determine the optimal number of topics with favorable model performance, we used a coherence score measure the modeling performance. Based on the performance as well as the meaning of the topics, we selected the final list of the topics.

### Data preparation and preprocessing

We collected the Tweets data from a public Twitter dataset, which contained daily Tweets data related to COVID-19.^17^ We used Twitter-API to get raw text Tweets and their corresponding user profiles. We retained Tweets related to COVID-19 vaccines using keywords and removed non-US Tweets based on user location information. Vaccine-related Tweets were collected by detecting keywords on the topic of the vaccine such as “vaccine” and “vaccination”. We also tracked information such as the number of Tweets and followers of each user to identify ghostwriters, who have unusual Tweet patterns. After obtaining the vaccine-related Tweets data, we processed the text data by removing irrelevant content for this study such as links. To train and test the sentiment analysis model, we randomly selected 2500 Tweets for annotation. Then, two classification models based on BERT were trained and selected to filter irrelevant Tweets and predict sentiment states. During the training process, we used back-translation for data augmentation.^33^ After training, these two classification models would be applied to all the Tweets data.

After data collection, we processed the data by removing redundant contents and potential noises. The specific dataset collection and preprocessing were as following steps:

- The data used in this study were sourced from a publicly available dataset, which has been collecting Tweets related to COVID-19 since January 1, 2020. We followed the instructions provided in the dataset's GitHub repository to access and download both the Tweets and user profiles associated with each Tweet ID, using Tweepy.^17^
- After downloading the raw data, Tweets posted by US users were selected based on the user location.
- Next, Tweets that did not have vaccine-related content or topics were dropped.
- By using the data obtained based on the above steps, the number of Tweet posts per day was calculated and used to filter users with unusual Tweet pattern (eg, ghostwriter). The relevant code and analyses are available at: https://github.com/haidog-yaqub/Vaccination_Sentiment.

### Data annotation

After obtaining vaccine-related Tweets data, to train a sentiment analysis model, we annotated a total of 2500 Tweets in the following steps: (1) in order to avoid the bias caused by topics that changed over time, we randomly selected 100 Tweets for each month from January 2021 to February 2022 (*n* = 1400 in total); (2) two authors (JY and JH) individually annotated 700 Tweets into four categories: positive, negative, irrelevant, and unclear (eg, neutral sentiment), (3) the research team discussed the annotation results and finally annotated these Tweets into three categories: positive, negative, and irrelevant. We only selected the Tweets that expressed a clear sentiment (positive or negative) for the follow-up analyses.

### Data augmentation

Tweet texts sometimes have formats like abbreviations, misspellings, etc. Meanwhile, relying on human intelligence to analyze a large amount of text data and train a robust model would not be feasible. To solve these issues, we used back-translation approach to augment the annotated data. With the effective application of deep learning translation models in sentence-level translation, translation tools could translate lots of languages into each other with promising performance; and they were also robust to handle typos and abbreviations.^34^ We leveraged the Google Translate API to perform this task, which could translate more than 130 languages.^35^

We employed the back-translation strategy in the following steps. First, we categorized all the available languages into language families based on information provided by Wikipedia. According to the populations of native speakers of each language family, we further selected five intermediate languages (Chinese, German, French, Russian, and Japanese) of the most used language families. Finally, a Tweet was translated into the target intermediate languages iteratively for five rounds, and the translated sentences were then translated back into English. To improve training efficiency, the back-translation was done before training and all data were saved locally. By randomly selecting 500 samples for manual verification, we found that back-translation not only enriched vocabulary and syntax, but also addressed the problems of colloquial expressions and typos. During the training process, we switched original Tweets and back-translated Tweets with different intermediate languages for each epoch. According to the testing results, the back-translation improved the model performance (F1-score) by more than 5%.

### Model training and applications

**Figure 2.**
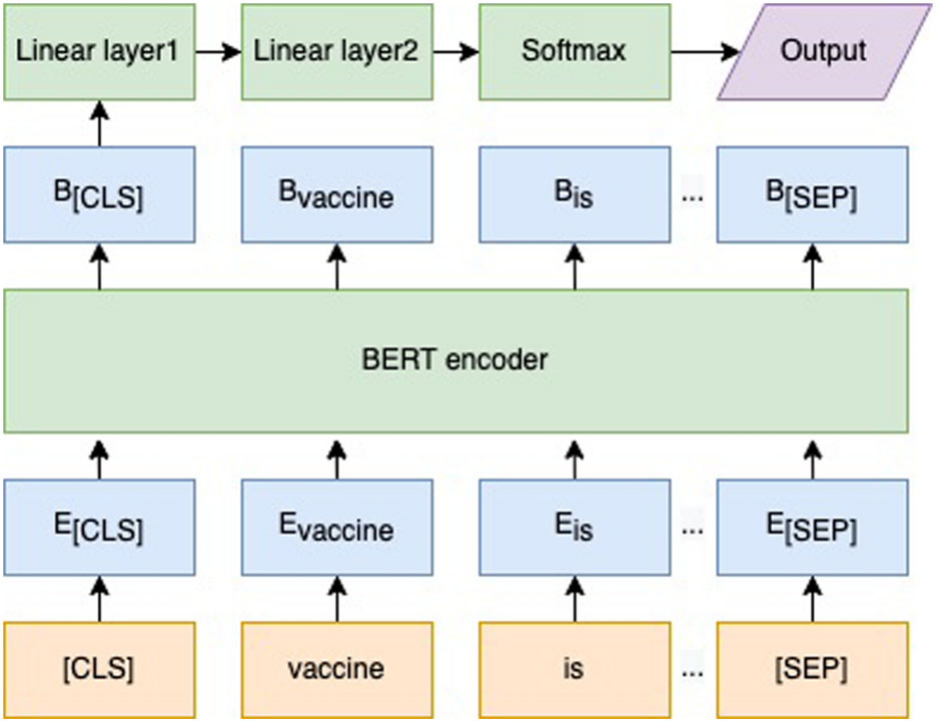
The framework of the sentiment analysis model.

As the labeled dataset by human intelligence was not sufficient to support the training of the sentiment from the scratch, we took advantage of transfer learning to complete the task. In the earlier NLP research, pretrained language models were usually used as feature extractors to obtain vector representations of words; then machine learning models were trained with these embeddings. Later, with the remarkable breakthroughs in deep learning models, the parameters of a pretrained model could be fine-tuned by retraining the model on a downstream task. Recently, transformer-based models like BERT achieved state-of-the-art performances on different kinds of downstream tasks such as text classification and sentiment analysis.^19^ To transfer the pretrained model to the sentiment task, we modified the torch-version pretrained BERT model provided by Hugging Face by using the [CLS] token as input to a fully connected network with one hidden layer, and the softmax activation function as the last layer of the model to perform classification.^36^ The analysis task was divided into two binary classification tasks: irrelevant content detection and sentiment analysis (Figure 2). The [CLS] was used to capture global features of the whole input text, and the [SEP] token helped the model to separate sentences. The E. denotes the embedding of each word, and the B. denotes the feature encoded by the BERT encoder.

We conducted our experiments on an NVIDIA GTX1070 TI GPU. The hyperparameters settings for the training were:

- Epochs = the number of back-translation languages × 3
- Batch size = 4
- Learning rate = 1e^−5^
- Weight decay = 1e^−5^
- Max sequence length = 512

As the annotated data might not be balanced, we considered 80% of each category as training data to fine-tune the weights of the pretrained model, 10% as validation data to select the best-performed model for the training, and 10% as testing data to evaluate the performance of the final models. Finally, the irrelevant content detection achieved 81.26% accuracy, and the sentiment analysis model achieved 79.67% accuracy and 87.53% F1 score on the testing dataset.

Next, we applied these two training models to the whole dataset. We filtered Tweets that were predicted as irrelevant content. The rate of Tweets with a negative sentiment each day was then calculated. We utilized the users’ location information to evaluate the geospatial variations among the states and temporal variations in public sentiments toward COVID-19 vaccination. We identified, evaluated, and associated the key events that impacted the positive or negative sentiments to the temporal trends. We also conducted qualitative syntheses of Tweets on points of interest to identify underlying themes and validated insights from the Tweets. We classified the 2500 randomly selected Tweets, discussed the themes, and mapped them on Levesque's model,^37^ which was designed to explain the comprehensiveness and dynamic nature of access to health care with five domains of accessibility (Approachability, Acceptability, Availability and accommodation, Affordability, and Appropriateness). We further illustrated the themes and provided examples within each of the domains.

## RESULTS

A total of 3 198 686 Tweets met our inclusion criteria. After applying the vaccination classifier model and opinion expression classifier model, 839 903 Tweets that were not relevant to COVID-19 vaccination or did not express an unclear sentiment were removed. The remaining 2 358 783 Tweets were then analyzed using sentiment analysis and topic models. Among the 2 358 783 Tweets, 824 755 (35.0%) expressed negative opinions towards vaccination while 1 534 028 (65.0%) demonstrated positive opinions. We further identified 1 738 550 (73.7%) Tweets that had hashtags (ie, “#”) within their content, which were used to analyze the topic.

Table 1 demonstrates major categories of reasons or concerns for opposing COVID-19 vaccination. The main barrier themes include accessibility, hesitancy, dislike forcing, safety concern, mistrust, manufacturing delays, inequity, conspiracy theory, and misinformation. Among the barriers, mistrust (31.8%), hesitancy (27.9%), and safety concern (20.3%) are the top three themes. Some individuals declined in trust of expertise and authority, and different modes of belief-based extremism.^38^ Political polarization, as well as libertarian views and alternative health care advocacy, triggered public questioning about the importance, safety, and effectiveness of COVID-19 vaccines.^39^ In addition, manufacturing delays also increased the negative sentiment toward vaccination.^40^ For example, in February 2021, vaccine distribution was disrupted in several states, including Texas, Missouri, Alabama, and New Hampshire due to severe winter storms.^41^

**Table 1.** A taxonomy of reasons or concerns for opposing COVID-19 vaccine or vaccination based on the Levesque's model

Domains	Definition	Barrier themes, (%)	Example Tweets
Approachability	People facing health needs can actually identify that some form of vaccine-related services exist, can be reached, and have an impact on the health of the individual.	Accessibility (1.4)	*Severe winter weather in parts of the U.S. is impacting COVID-19 vaccinations, delaying vaccine deliveries and appointments.*
Acceptability	Cultural and social factors determining the possibility for people to accept the aspects of the vaccine and the judged appropriateness for the persons to seek the vaccine.	Hesitancy (27.9) Dislike forcing (8.6) Safety concern (20.3) Mistrust (31.8%)	*The flu vaccines are derived from the flu virus. They were never able to isolate the coronavirus and as such, are completely unable to create a vaccine from it. It's NOT a vaccine no matter how many people keep calling it that.* *What's the point of a mandate? Anyone that wants a Covid-19 vax can get one. If someone chooses not to, why force them? If the vaccines work, the mandates can't be to protect the vaxxed.* *Severe breakthrough cases are rare but possible. And research suggests that those who are more vulnerable to COVID-19, in general, are also more at risk for a severe case after vaccination.* *For everyone refusing a vaccine because it is only FDA “authorized”, not “approved”. Guess What, Regeneron Monoclonal Antibodies are also ONLY FDA “authorized”, not “approved”*
Availability and accommodation	Vaccination services (either the physical space or those working in health care roles) can be reached both physically and in a timely manner.	Manufacturing delays (1.4)	*Manufacturing delays are slowing production of Johnson & Johnson’s one-shot #COVID19 vaccine.*
Affordability	Economic capacity for people to spend resources and time to use appropriate vaccination services.	Inequity (4.4)	*Let's be really clear; the virus doesn't care about your ZIP code. This uptake disparity reflects both access and legacy inequities (who do you trust?), and we'll have many more waves until this is addressed.*
Appropriateness	The fit between vaccination services and clients’ needs, its timeliness, the amount of care spent in assessing health problems and determining the correct treatment, and the technical and interpersonal quality of the vaccination services provided.	Conspiracy theory (0.7) Misinformation (3.5)	*CO=covid19 or coronavirus V=vaccination ID=identification 1 = A 9 = I. Covid19 vaccination ID-AI. The id is the way to move the west into social credit scores of CCP and manage the data through AI to control us.* *Instagram has banned Robert F Kennedy Jr for making false claims about coronavirus and vaccines. Unfortunately, history shows that vaccine misinformation is harder to stamp out than you think.*

**Figure 3.**
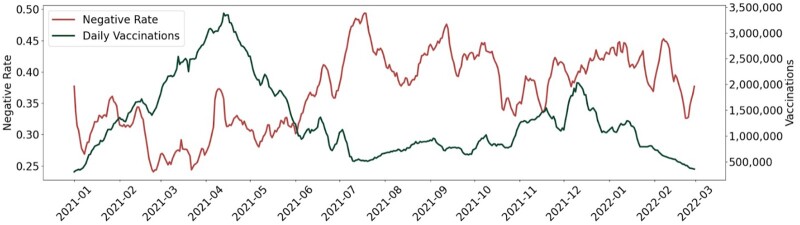
Weekly rolling average time-series results of Tweets sentiments (left Y-axis) and vaccinations (right Y-axis).

Figure 3 demonstrates the weekly rolling average time-series results of Tweets sentiments (red line) and vaccinations (green line). Because some states did not administer vaccines during the weekend, we used the weekly rolling average rather than daily measures. Overall, the two lines present opposite tendencies over time. There were generally more Tweets that held positive opinions on vaccination. There was a steady increase in vaccination since January 2021 and reached the peak in April 2021 and followed by a sharp decrease after April 2021.

**Figure 4.**
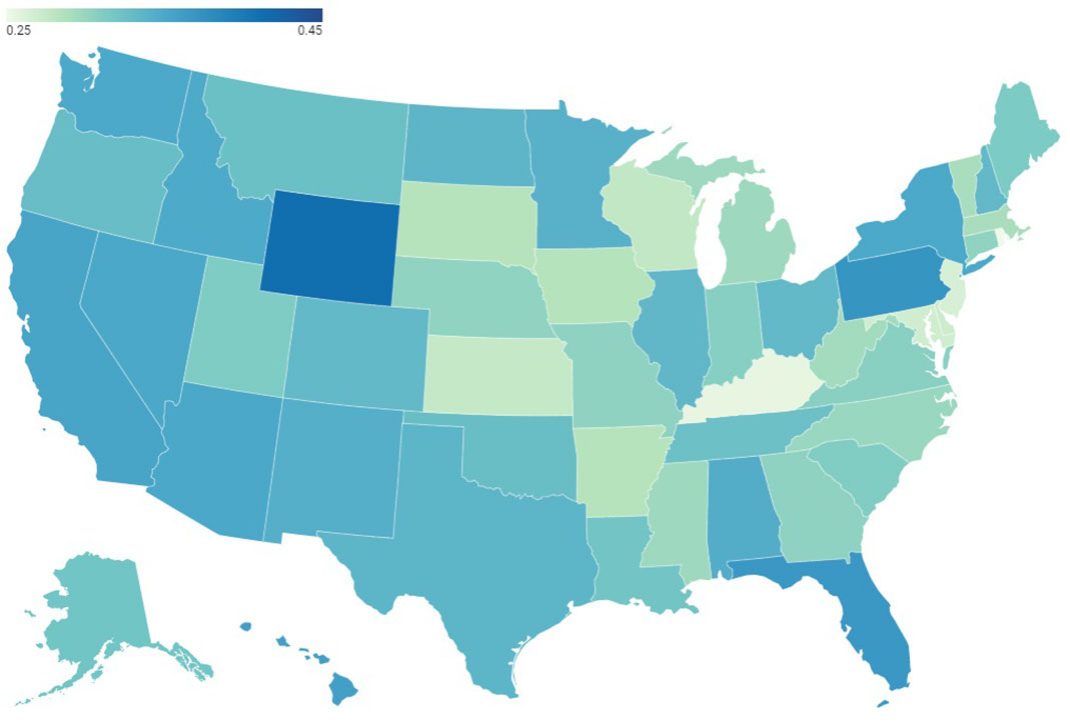
Geospatial mapping of negative sentiments on Tweets in the United States toward the COVID-19 vaccination.

Figure 4 presents the geospatial mapping of Twitter negative sentiments in the United States toward the COVID-19 vaccine. A geospatial map of overall (averaged) sentiments at the state level indicates that most states had a moderate positive sentiment. The states with relatively higher negative sentiment toward COVID-19 vaccination were concentrated in the west, and some states in the east and southeast regions, the top five states of negative sentiment include Wyoming, Pennsylvania, Florida, Hawaii, and California.

**Figure 5.**
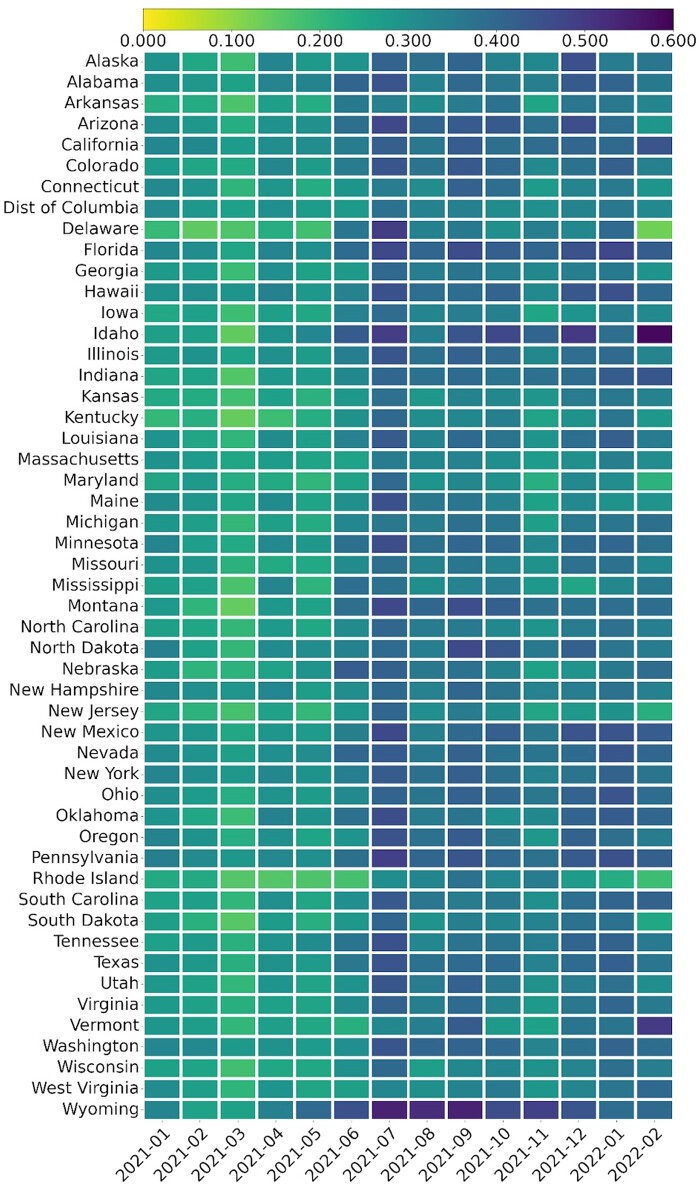
Negative sentiments on Tweets in each state of the United States toward the COVID-19 vaccination over time (sorted by alphabetical).

Figure 5 presents the negative sentiments in each state of the United States toward the COVID-19 vaccine over time. As time went by, the negative sentiment rate increased and reached the highest in July 2021, which aligned with the results in Figure 2. We also see variabilities across the states.

**Figure 6.**
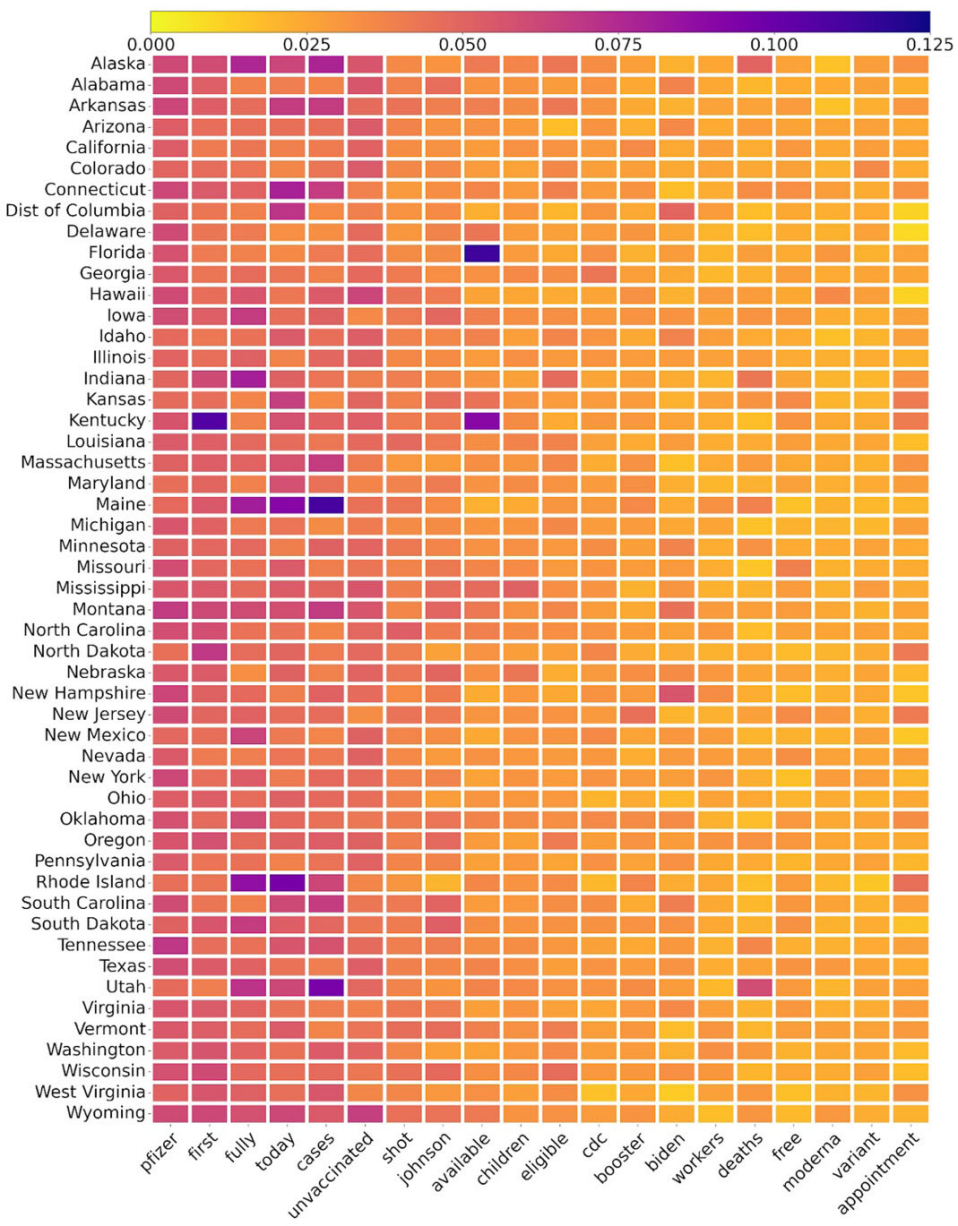
Major words rate on Tweets in each state of the United States toward the COVID-19 vaccination (sorted by alphabetical). Numerator: the word’s frequency in the state; denominator: the total number of Tweets in the state.

Figure 6 presents major word rates in each state of the United States regarding the COVID-19 vaccine. The numerator is the word’s frequency in the state and the denominator is the total number of Tweets in the state. The top 10 words are Pfizer, first, fully, today, cases, unvaccinated, shot, Johnson, available, and children. We see moderate variation across the states, but some words were substantial in some states. For example, “available” has a high rate in Florida; “first” and “available” have high rates in Kentucky; “fully”, “today”, and “cases” have high rates in Maine, Rhode Island, and Utah. The word “first” and “fully” were always linked with the “first does” and “fully vaccinated” Tweets; the word “today” was identified and included in the major word list because it was related the dissemination of the COVID-19 vaccine availability and callout of getting vaccinated.

**Figure 7.**
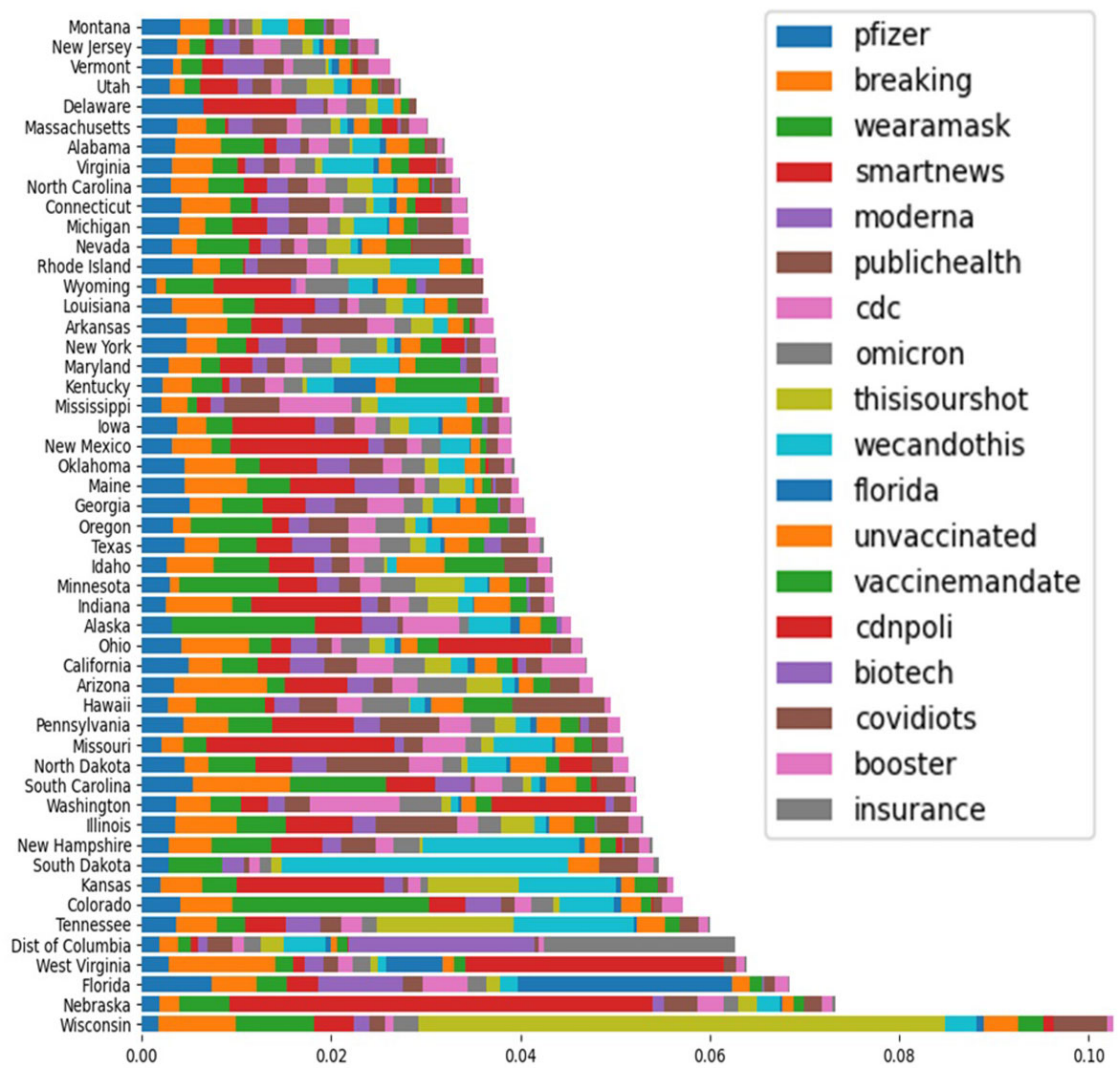
Major hashtag-based topic rate on Tweets in each state of the United States toward the COVID-19 vaccination (sorted by topics’ rate).

Figure 7 presents the major hashtag-based topic rates on Twitter in each state of the United States toward COVID-19 vaccination. The numerator is the topic’s frequency in the state and the denominator is the total number of Tweets in the state. Among the selected 18 topics, Wisconsin has the highest total rate, which means the COVID-19 vaccine-related Tweets that residents in Wisconsin posted contained most of these selected topics. In addition, the majority of relevant Tweets in Wisconsin contained “thisisourshot”, which meant this campaign gained a good buy-in in Wisconsin. The top 10 topics are Pfizer, breaking, wearamask, smartnews, moderna, publichealth, cdc, omicron, thisisourshot, and wecandothis. Only “Pfizer” is the only same topic as the results of the major words rate in Figure 6, which means the Pfizer vaccine gained the most popularity on the Twitter platform.

## DISCUSSION

Social media like Twitter provides the opportunity to collect data related to vaccination in nearly real-time. This digital platform also allows new methods of analysis and the opportunity to investigate the effect of the sentiment on vaccine uptake.^42^ In this study, we analyzed public opinions expressed on Twitter regarding COVID-19 vaccination in the United States. We studied a total of 3 198 686 Tweets that were collected from January 2021 to February 2021 using an analytical strategy that combined qualitative content analysis^43^ for understanding opinions expressed in subtle human language and machine learning for scalability. Comparative analysis revealed that over the 14-month study period, the overall public sentiment toward COVID-19 vaccination was moderate positive. The top three barriers to vaccination were mistrust, hesitancy, and safety concerns.

The sentiment across the states showed manifest variability. There are some potential reasons for this pattern: (1) In April 2021, the United States surpassed 200 million vaccinations administered, most of whom might hold positive sentiment toward vaccination; individuals who posted Tweets related to vaccines might be the remaining population who held negative sentiment; (2) CDC and FDA paused the use of the Johnson and Johnson COVID-19 vaccine because of the blood clot complications; Moderna vaccine also underwent a similar dynamic in terms of concerns^44^; these events might increase individuals’ concerns about vaccine safety; (3) In June 2021, the Delta variant, which was first identified in India in late 2020, became the dominant variant in the United States. The variant kicked off a third wave of infections during the summer of 2021, which might decrease individuals’ outdoor activities and caused the decreased vaccination. Since the fall of 2021, many states issued vaccine mandates policy. This policy likely increased the vaccinations between October 2021 and December 2021. In the middle of December 2021, the number of daily vaccinations decreased, and the reasons might be: (1) the number of fully vaccinated approached a moderately saturated state; according to the real-time data, more than 83% of individuals had at least one dose and more than 71% individuals were fully vaccinated.^45^

The early detection of an opinion shift might be useful in the context, in which many countries from around the world have been working on the COVID-19 vaccination, as it would promote actions aimed at increasing the general public’s confidence towards vaccination. Many people search online for health-related information and the information will impact patient decision-making; it is therefore essential to understand what is shared online.^46^

The results demonstrate the potential of NLP-based real-time social media monitoring of public sentiments and attitudes to help detect and prevent vaccination hesitancy and concerns. This monitoring may inform more effective strategies for vaccine deployment, including decisions on prioritization and equitability, to help maximize the uptake of the vaccines.^43^

Given the dramatic changes in the communication landscape that fuel the rapid spread of vaccine information alongside misinformation, new methodologies are needed to monitor emerging vaccine concerns over time and place in order to better inform appropriate responses.^47^^,^^48^ We analyzed the temporal variations in public sentiments toward COVID-19 vaccines in the United States. We identified, evaluated, and mapped the keywords and hashtag-based topics impacting positive and negative sentiments to the temporal trends. Mapping vaccine hesitancy at a local level is also one important step towards addressing it, along with other needed interventions at the individual and community levels. We mapped spatial variations in public sentiment to regions in the United States. The geospatial maps could help identify areas with more negative sentiments toward COVID-19 vaccination, which can be further studied for potential interventions to allay the underlying public concerns.^49^ We found that different states presented various trend patterns in sentiment change over time. Geospatial analysis and map visualization can better portray more aspects of residents’ attitudes towards COVID-19 vaccination, which may be helpful for government and public health agencies to conduct COVID-19 vaccination campaigns in areas that need more attention and efforts to address the barriers and concerns. The large volume of timely data on social media has provided an opportunity to develop spatially detailed estimates of vaccination sentiment (ie, mapping by location).^50^ Spatially refined estimates of vaccination sentiment have proved to be useful in local efforts to increase vaccination rates.^51^ The information may be used by community-based programs to tailor their efforts to local areas that have the greatest need.^52^^,^^53^ The geographical patterns can also be used to identify places to provide mobile vaccination clinics and initiate measures for reducing barriers to vaccination. Local information can also be used to monitor the effectiveness of local interventions, including the effect of various types of vaccination mandates. Furthermore, there have been cases where vaccine debates that are purposefully polarized, thus exploiting the doubting public and system weaknesses for political purposes, while waning vaccine confidence elsewhere may be influenced by a general distrust in the government and scientific elites.

### Limitation

This study has several limitations. First, social media data are likely to be biased as the users may be younger and from more urbanized areas. These intrinsic characteristics of social media users may influence the results. In addition, the results cannot provide causality of the variations in the social media sentiments. However, the results are potentially helpful for monitoring progress toward vaccination equity. Second, manual classifications of Tweets for the “ground-truth” testing data annotation were based on the research team’s understanding and interpretations of the tweet, which might introduce errors. However, this study focused on the positive and negative tweet, and we excluded Tweets that were with irrelevant and unclear sentiment when conducting the data annotation, which improved the accuracy of the following analyses. Future work are needed to develop diverse and effective machine learning classifiers to facilitate opinion mining using social media data and the automatic and continuous extraction and monitoring of public opinions. Large and complex datasets on vaccination should also be analyzed according to other identifiers such as a granular geospatial unit (eg, Zip code) and individual characteristics, including social determinants of health, which can help to advance further microtarget vaccine deployment efforts.^54^ In addition, future work should include more diverse social media platforms representing different types of user groups, different interaction modalities, and geographic settings to address health care disparity.

## CONCLUSION

This study demonstrates the potential of an analytical pipeline, which integrates NLP-enabled modeling, time series, and geospatial analyses of social media data. Through the analysis of a large Twitter dataset using a combination of NLP and qualitative content analysis, we classified the public’s attitude toward COVID-19 vaccination, the temporal trend over time, and geographic sentiment distribution. The results showed that while generally more Tweets held positive opinions on vaccination, negative opinions were not uncommon. The sentiment towards vaccination across the states showed manifest variability. The top three barriers to vaccination were mistrust, hesitancy, and safety concerns. The resilience of vaccination programs may be influenced by the rapid and global spread of misinformation. Public confidence in COVID-19 vaccines can be exacerbated by unproven concerns regarding vaccine safety, which seed doubt and mistrust. The NLP-enabled real-time social media monitoring of public sentiments and attitudes can help detect public sentiment towards COVID-19 vaccination, which may help solution providers to understand the reasons why some social groups may be reluctant to be vaccinated against COVID-19. The results could provide support for developing tailored policies, interventions, and implementation strategies to facilitate COVID-19 vaccination.

## Data Availability

All data referred to in the manuscript are publicly available at: https://github.com/thepanacealab/covid19_twitter. The relevant code and analyses are available at: https://github.com/haidog-yaqub/Vaccination_Sentiment.

## References

[1] US FDA. Comirnaty and Pfizer-BioNTech COVID-19 Vaccine. Maryland: The United States Food and Drug Administration; 2021.

[2] Ye J , Wang Z, Hai J. Social networking service, patient-generated health data, and population health informatics: national cross-sectional study of patterns and implications of leveraging digital technologies to support mental health and well-being. J Med Internet Res 2022; 24 (4): e30898. 35486428 10.2196/30898 PMC9107051

[3] Ye J , Li N, Lu Y, Cheng J, Xu Y. A portable urine analyzer based on colorimetric detection. Anal Methods 2017; 9 (16): 2464–71.

[4] Ye J , Yao L, Shen J, et al Predicting mortality in critically ill patients with diabetes using machine learning and clinical notes. BMC Med Inform Decis Mak 2020; 20 (S11): 1–7. 33380338 10.1186/s12911-020-01318-4 PMC7772896

[5] Ye J , Sanchez-Pinto LN. Three data-driven phenotypes of multiple organ dysfunction syndrome preserved from early childhood to middle adulthood. In: AMIA annual symposium proceedings. American Medical Informatics Association; 2020: 1345. PMC8075454 33936511

[6] Ye J , Ren Z. Examining the impact of sex differences and the COVID-19 pandemic on health and health care: findings from a national cross-sectional study. JAMIA Open 2022; 5 (3): ooac076. 10.1093/jamiaopen/ooac076 PMC9494404 36177395

[7] Wang J , Abu-el-Rub N, Gray J, et al COVID-19 SignSym: a fast adaptation of a general clinical NLP tool to identify and normalize COVID-19 signs and symptoms to OMOP common data model. J Am Med Inform Assoc 2021; 28 (6): 1275–83. 33674830 10.1093/jamia/ocab015 PMC7989301

[8] Schöning V , et al Automatic identification of risk factors for SARS-CoV-2 positivity and severe clinical outcomes of COVID-19 using Data Mining and Natural Language Processing. medrXiv, 2021. 10.1101/2021.03.25.21254314

[9] Low DM , Rumker L, Talkar T, et al Natural language processing reveals vulnerable mental health support groups and heightened health anxiety on reddit during covid-19: observational study. J Med Internet Res 2020; 22 (10): e22635. 32936777 10.2196/22635 PMC7575341

[10] Ye J. Pediatric mental and behavioral health in the period of quarantine and social distancing with COVID-19. JMIR Pediatr Parent 2020; 3 (2): e19867. 32634105 10.2196/19867 PMC7389340

[11] Fernandes M , Sun H, Jain A, et al Classification of the disposition of patients hospitalized with COVID-19: reading discharge summaries using natural language processing. JMIR Med Inform 2021; 9 (2): e25457. 33449908 10.2196/25457 PMC7879729

[12] Kwon J , Grady C, Feliciano JT, et al Defining facets of social distancing during the COVID-19 pandemic: Twitter analysis. J Biomed Inform 2020; 111: 103601. 33065264 10.1016/j.jbi.2020.103601 PMC7553881

[13] Ye J. Advancing mental health and psychological support for health care workers using digital technologies and platforms. JMIR Form Res 2021; 5 (6): e22075. 34106874 10.2196/22075 PMC8274671

[14] Memon SA , Carley KM. Characterizing covid-19 misinformation communities using a novel twitter dataset. arXiv, arXiv:2008.00791, 2020, preprint: not peer reviewed.

[15] WHO. Ten threats to global health in 2019. 2019.

[16] WHO. Data for action: achieving high uptake of COVID-19 vaccines: gathering and using data on the behavioural and social drivers of vaccination: a guidebook for immunization programmes and implementing partners: interim guidance, 3 February 2021, World Health Organization; 2021.

[17] Banda JM , Tekumalla R, Wang G, et al A large-scale COVID-19 Twitter chatter dataset for open scientific research – an international collaboration. Epidemiologia 2021; 2 (3): 315–24. 36417228 10.3390/epidemiologia2030024 PMC9620940

[18] Tekumalla R , Banda JM. Social media mining toolkit (SMMT). Genomics Inform 2020; 18 (2): e16. 32634870 10.5808/GI.2020.18.2.e16 PMC7362951

[19] Devlin J , et al BERT: pre-training of deep bidirectional transformers for language understanding. arXiv, arXiv:1810.04805, 2018, preprint: not peer reviewed.

[20] General Data Protection Regulation. Intouch, 2018: 25.

[21] Gellert R. Understanding the notion of risk in the General Data Protection Regulation. Comput Law Secur Rev 2018; 34 (2): 279–88.

[22] Neri F , Aliprandi C, Capeci F, Cuadros M. Sentiment analysis on social media. In: 2012 IEEE/ACM international conference on advances in social networks analysis and mining. IEEE; 2012: 919–26.

[23] Yates TM , Dodds MF, Sroufe LA, et al Exposure to partner violence and child behavior problems: a prospective study controlling for child physical abuse and neglect, child cognitive ability, socioeconomic status, and life stress. Dev Psychopathol 2003; 15 (1): 199–218. 12848442 10.1017/s0954579403000117

[24] Lang P , Bradley MM. The International Affective Picture System (IAPS) in the study of emotion and attention. In: Coan JA, Allen JJB, eds. Handbook of Emotion Elicitation and Assessment. London: Oxford University Press; 2007, Vol. 29: 70–3.

[25] Medhat W , Hassan A, Korashy H. Sentiment analysis algorithms and applications: a survey. Ain Shams Eng J 2014; 5 (4): 1093–113.

[26] Feldman R. Techniques and applications for sentiment analysis. Commun ACM 2013; 56 (4): 82–9.

[27] Agarwal A , Xie B, Vovsha I, Rambow O, Passonneau RJ. Sentiment analysis of twitter data. In: proceedings of the workshop on language in social media (LSM 2011); 2011: 30–8.

[28] Zhang L , Wang S, Liu B. Deep learning for sentiment analysis: a survey. Wiley Interdiscip Rev Data Mining Knowledge Discov 2018; 8 (4): e1253.

[29] Ain QT , Ali M, Riaz A, et al Sentiment analysis using deep learning techniques: a review. Int J Adv Comput Sci Appl 2017; 8 (6).

[30] Sun C , Huang L, Qiu X. Utilizing BERT for aspect-based sentiment analysis via constructing auxiliary sentence. arXiv, arXiv:1903.09588, 2019, preprint: not peer reviewed.

[31] Chang HC. A new perspective on Twitter hashtag use: diffusion of innovation theory. Proc Am Soc Inform Sci Technol 2010; 47(1): 1–4.

[32] Deerwester S , Dumais ST, Furnas GW, et al Indexing by latent semantic analysis. J Am Soc Inf Sci 1990; 41 (6): 391–407.

[33] Xie Q , et al Unsupervised data augmentation for consistency training. Adv Neural Inform Process Syst 2020; 33: 6256–68.

[34] Farzindar AA , Inkpen D. Natural language processing for social media. In: Synthesis Lectures on Human Language Technologies; 2020, Vol. 13: 1–219.

[35] Turovsky B. See the world in your language with Google Translate. Google, 2015. https://blog.google/products/translate/see-world-in-your-language-with-google/.

[36] Wolf T , et al Huggingface's transformers: state-of-the-art natural language processing. arXiv, arXiv:1910.03771, 2019, preprint: not peer reviewed.

[37] Levesque J-F , Harris MF, Russell G. Patient-centred access to health care: conceptualising access at the interface of health systems and populations. Int J Equity Health 2013; 12 (1): 18–9. 23496984 10.1186/1475-9276-12-18 PMC3610159

[38] Jennings W , Stoker G, Bunting H, et al Lack of trust, conspiracy beliefs, and social media use predict COVID-19 vaccine hesitancy. Vaccines 2021; 9 (6): 593. 34204971 10.3390/vaccines9060593 PMC8226842

[39] Dolman AJ , Fraser T, Panagopoulos C, Aldrich DP, Kim D. Opposing views: associations of political polarization, political party affiliation, and social trust with COVID-19 vaccination intent and receipt. J Public Health 2023; 45 (1): 36–9. 10.1093/pubmed/fdab401 PMC9383304 35077546

[40] Ye J , Zhang R, Bannon JE, et al Identifying practice facilitation delays and barriers in primary care quality improvement. J Am Board Fam Med 2020; 33 (5): 655–64. 32989060 10.3122/jabfm.2020.05.200058

[41] CDC. CDC Museum COVID-19 Timeline. Centers for Disease Control and Prevention, 2022. https://www.cdc.gov/museum/timeline/covid19.html.

[42] Muric G , Wu Y, Ferrara E. COVID-19 vaccine hesitancy on social media: building a public twitter data set of antivaccine content, vaccine misinformation, and conspiracies. JMIR Public Health Surveill 2021; 7 (11): e30642. 34653016 10.2196/30642 PMC8694238

[43] Ye J , Woods D, Bannon J, et al Identifying contextual factors and strategies for practice facilitation in primary care quality improvement using an informatics-driven model: framework development and mixed methods case study. JMIR Hum Factors 2022; 9 (2): e32174. 35749211 10.2196/32174 PMC9269526

[44] Marks P , Schuchat A. Joint CDC and FDA Statement on Johnson & Johnson COVID-19 Vaccine. US Food & Drug Administration, 2021.

[45] Vaccines for COVID-19. https://www.cdc.gov/coronavirus/2019-ncov/vaccines/index.html. Accessed 2019.

[46] Kata A. Anti-vaccine activists, Web 2.0, and the postmodern paradigm – an overview of tactics and tropes used online by the anti-vaccination movement. Vaccine 2012; 30 (25): 3778–89. 22172504 10.1016/j.vaccine.2011.11.112

[47] Fridman A , Gershon R, Gneezy A. COVID-19 and vaccine hesitancy: a longitudinal study. PLoS ONE 2021; 16 (4): e0250123. 33861765 10.1371/journal.pone.0250123 PMC8051771

[48] Ye J. Health information system's responses to COVID-19 pandemic in China: a national cross-sectional study. Appl Clin Inform 2021; 12 (02): 399–406. 34010976 10.1055/s-0041-1728770 PMC8133837

[49] Ye J. The role of health technology and informatics in a global public health emergency: practices and implications from the COVID-19 pandemic. JMIR Med Inform 2020; 8 (7): e19866. 32568725 10.2196/19866 PMC7388036

[50] Ye J. The impact of electronic health record – integrated patient-generated health data on clinician burnout. J Am Med Inform Assoc 2021; 28 (5): 1051–6. 33822095 10.1093/jamia/ocab017 PMC8068436

[51] Collis A , Garimella K, Moehring A, et al Global survey on COVID-19 beliefs, behaviours and norms. Nat Hum Behav 2022; 6 (9): 1310–7. 35606513 10.1038/s41562-022-01347-1

[52] Ye J. Design and development of an informatics-driven implementation research framework for primary care studies. *AMIA Annu Symp Proc* 2022; 2021: 1208–14. PMC8861697 35308925

[53] Ye J , Orji IA, Baldridge AS, et al, Hypertension Treatment in Nigeria Program Investigators. Characteristics and patterns of retention in hypertension care in primary care settings from the hypertension treatment in Nigeria Program. JAMA Netw Open 2022; 5 (9): e2230025. 36066896 10.1001/jamanetworkopen.2022.30025 PMC9449788

[54] Ye J , Ma Q. The effects and patterns among mobile health, social determinants, and physical activity: a nationally representative cross-sectional study. *AMIA Jt Summits Transl Sci Proc* 2021; 2021: 653–62. PMC8378627 34457181

